# PepSim: T-cell cross-reactivity prediction via comparison of peptide sequence and peptide-HLA structure

**DOI:** 10.3389/fimmu.2023.1108303

**Published:** 2023-04-28

**Authors:** Sarah Hall-Swan, Jared Slone, Mauricio M. Rigo, Dinler A. Antunes, Gregory Lizée, Lydia E. Kavraki

**Affiliations:** ^1^ Department of Computer Science, Rice University, Houston, TX, United States; ^2^ Department of Biology and Biochemistry, University of Houston, Houston, TX, United States; ^3^ Department of Melanoma Medical Oncology, University of Texas MD Anderson Cancer Center, Houston, TX, United States

**Keywords:** T-cell cross-reactivity, peptide-HLA, immunotherapy, structure comparison, sequence similarity

## Abstract

**Introduction:**

Peptide-HLA class I (pHLA) complexes on the surface of tumor cells can be targeted by cytotoxic T-cells to eliminate tumors, and this is one of the bases for T-cell-based immunotherapies. However, there exist cases where therapeutic T-cells directed towards tumor pHLA complexes may also recognize pHLAs from healthy normal cells. The process where the same T-cell clone recognizes more than one pHLA is referred to as T-cell cross-reactivity and this process is driven mainly by features that make pHLAs similar to each other. T-cell cross-reactivity prediction is critical for designing T-cell-based cancer immunotherapies that are both effective and safe.

**Methods:**

Here we present PepSim, a novel score to predict T-cell cross-reactivity based on the structural and biochemical similarity of pHLAs.

**Results and discussion:**

We show our method can accurately separate cross-reactive from non-crossreactive pHLAs in a diverse set of datasets including cancer, viral, and self-peptides. PepSim can be generalized to work on any dataset of class I peptide-HLAs and is freely available as a web server at pepsim.kavrakilab.org.

## Introduction

The cellular immune response is a vital part of our defense mechanism against various diseases, including viral infection and cancer. As part of this immune response, cytotoxic T-cells are specialized to defend against specific diseases by pinpointing and eliminating infected cells. This occurs *via* interaction of the T-cell receptor (TCR) with the peptide-human leukocyte antigen class I (pHLA) complex on the surface of the target cells (1). The pHLA is formed when HLA receptors bind to peptides inside the cell and display them on the cell surface. TCRs are specialized to recognize pHLAs when the peptide that is being presented is not normally produced by the cell, which means that the T-cell can respond against non-self peptides.

An intrinsic feature of T-cells is cross-reactivity, which refers to the natural ability of a TCR to recognize more than one pHLA (2). In the context of a viral infection, cross-reactivity allows for a broader response of a single T-cell against multiple viral targets (e.g., variants of the same virus or related viruses) (3). However, the broader specificity caused by cross-reactivity can be the source of dangerous off-target toxicity in the context of cancer immunotherapy (e.g., T-cell-based immunotherapy) (4). One method of immunotherapy is adoptive T-cell transfer, where a large number of tumor-specific T-cells are delivered to the patient to amplify the immune response against the tumor. Because of cross-reactivity, there exist cases where therapeutic T-cells directed towards specific tumor pHLA complexes may also recognize self-peptide-HLAs, causing autoimmune side effects (5, 6). Therefore, preventing T-cell cross-reactivity in these cases is critical for designing T-cell-based cancer immunotherapies that are both effective and safe.

T-cell cross-reactivity is driven by the similarity between pHLAs, but the nature of that similarity is not fully defined. Previous studies have shown that peptide sequence similarity is not sufficient in all cases to predict T-cell cross-reactivity and highlighted the importance of pHLA structure and biochemical properties such as electrostatic potential (7–9). Previous computational works on T-cell cross-reactivity define similarity in different ways. For example, one method defines peptide sequence similarity as the number of identical amino acids at each position in the peptide (10). Because each position in a peptide is not equally important to TCR-pHLA binding, the authors also examined the experimentally determined structure of TCR-pHLA complexes available in the Protein Data Bank (11) to determine the positions of the peptide that are in contact with the TCR. Those positions that are in contact with the TCR are deemed “important” and therefore considered in the calculation of sequence similarity. A similar method is employed by JanusMatrix (12), a part of EpiVax’s proprietary immunogenicity screening kit. Focusing on what they define as the “TCR facing residues”, the authors define the similarity between peptides as identical amino acids. Additional methods of predicting T-cell cross-reactivity include RACER, a method of predicting TCR-pHLA binding affinity using supervised machine learning techniques (13). Also, iCrossR and Expitope use transcript and tissue abundance levels of peptide sequences to predict the likelihood of off-target toxicity (14, 15). Expitope 2.0 is available as a web server. Finally, a method developed by Antunes et al. (7) and later optimized by Mendes et al. (8) implicitly accounted for both structural information and biochemical features through the analysis of 2D images of the TCR-interacting surfaces of pHLAs (7, 8).

In this paper, we present PepSim, a novel computational method for calculating the similarity between pHLAs to predict T-cell cross-reactivity. Our method calculates a similarity score based on peptide sequence and 3D structural information. We focus the structural analysis on the region of the pHLA that interacts with the TCR, specifically analyzing the pHLA surface. We show that our score can differentiate between cross-reactive and non-cross-reactive pHLAs with high accuracy using five datasets of viral, cancer, or self-peptides. Each dataset includes peptides that were experimentally determined to be recognized by the same TCR.

We define a novel similarity score that is calculated between pHLAs. The input is a list of peptides and the structures of those peptides bound to the HLA. These structures can be crystal structures (i.e., from the Protein Data Bank (11)) or generated by modeling programs such as APE-Gen (16) or DockTope (17). We calculate the sequence similarity between peptides as well as the structural and biochemical similarity between pHLAs, once the peptide has been docked on the HLA. The output is a 2D matrix where element i,j is the similarity score between peptides (and corresponding pHLAs) i and j . A low score indicates higher similarity than a high score. The matrix can then be used to cluster the peptides. Peptides (and pHLAs) that are clustered together based on the similarity score are considered more likely to be cross-reactive. PepSim is available as a web server at pepsim.kavrakilab.org.

## Methods

### Sequence similarity

We calculate the sequence similarity between each pair of input peptides in three ways. The input for each method is the list of peptide sequences, and the output is a 2D matrix containing the pairwise similarity scores. The first sequence similarity score is calculated using a BLOSUM matrix, where each pair of amino acids is assigned an integer value based on the relative frequencies of amino acids (18). The BLOSUM62 matrix values are calculated based on amino acid sequence alignments with less than 62% identity. In our method, the similarity between two peptides is defined as the sum of the BLOSUM62 values for the amino acid pair at each position of the peptides, which is a common method of calculating sequence similarity. Secondly, we calculated the pairwise similarity between peptides using the similarity matrix calculated by HLAthena (19), which calculates the entropy at each peptide position based on the entire dataset and uses the entropy to weight the importance of each peptide position when calculating similarity based on the PMBEC similarity matrix. Lastly, we calculated the Hamming distance between two peptides, defined as the number of amino acid positions that differ between the two peptides (i.e., AAAA and AAAB have a Hamming distance of 1 because only position 4 differs). The combination of these three similarity metrics was empirically observed to give the best results.

### Structural and biochemical similarity

The pHLA pairwise similarity is calculated starting from a dataset of pHLA structures. The output is another 2D matrix of pairwise similarity scores. Depending on the source of the structures, their reference frame may be different, so we first align the structures using the align function of PyMOL (20). Then, we extract the solvent-accessible surface value from each structure using the program MSMS with a density of 3.0 and a probe size of 1.5Å (21). MSMS computes the surfaces as a triangular mesh, which we then downsample to a resolution of 1.0Åusing pymesh (22). We then annotate the vertices of this mesh with biochemical features, specifically the electrostatic potential, hydrophobicity, and hydrogen bond potential. The electrostatic potential of the surface is calculated using APBS (23). The hydrophobicity of each amino acid in the pHLA is assigned based on the Kyte-Doolittle scale (24), and assigned to each surface point based on the closest amino acid. Finally, the hydrogen bond potential at each point is calculated based on the free hydrogens of the closest amino acid residues. We calculate the hydrogen bond potential using the data preparation method of MaSIF (25), based on an orientation-dependent hydrogen bonding potential (26). In brief, the hydrogen bond potential at a vertex is calculated based on the vertex’s distance and angle from potential hydrogen donors (polar hydrogens) and potential acceptors (nitrogen or oxygen). The potential ranges between -1 (hydrogen bond acceptor) and +1 (hydrogen bond donor).

To account for the T-cell only interacting with a specific part of the pHLA complex, we define the TCR-interacting region as a round patch centered on the peptide bound to the HLA. The center of the patch is calculated by finding the closest surface point to the peptide’s center of mass. A circular patch is extracted by selecting the vertices in the neighborhood of the center point up to 16 edges away from the center, as defined by the triangular mesh. The surface patch mesh is converted to a point cloud where each point is a vertex from the mesh, and each point is annotated with the biochemical features.

To calculate the similarity between pHLAs, we first perform a pairwise alignment of the point clouds using the Iterative Closest Point (ICP) algorithm (27). This alignment uses only geometric information and does not take into account the annotated biochemical features. The ICP is an iterative procedure that aligns a source point cloud S to a target cloud T in 3D space by iterating over three main steps. The first is to create a corresponding point set C by matching points in the source point cloud to points on the target point cloud within a distance of ϵ=2 Å. By taking points within distance ϵ of each other, we account for the fact that there may be an unequal number of points in point clouds S and T . The psilon ball approach is a standard procedure in ICP (27). The second step is to calculate the rotation and translation that will best minimize the distance D between each corresponding point pair (i.e., to find the best transformation to align each source point to its corresponding target point). For a pair of point clouds S and T and the set of corresponding points C , the distance D is defined as


$$D=\sum_{i,j\in C}\|S_{i}-T_{j}{\|}_{2}$$

Where $S_{i}$ is the vector of x , y , and z coordinates of point i of point cloud S . The third step of ICP is to transform the source points using the rotation and translation found in the previous step. These three steps are repeated, recreating the corresponding point set for each iteration until convergence: when the change in D is less than 1.0e−06 , or until 30 iterations have been performed. ICP results in a final distance D and the indices of the corresponding points for each point cloud.

We define a new score function to calculate the similarity between aligned point clouds using the geometric coordinates and the biochemical features. We expand the ICP distance D so that each vertex in the point clouds has six dimensions, to include not only the geometric coordinates x , y , and z , but also the biochemical features electrostatic potential, hydrogen bond potential, and hydrophobicity. We also account for the size of the corresponding point set, as a low number of corresponding points indicates that the source and target point clouds are not well aligned. Our distance score $D_{2}$ is defined as


$$D_{2}=\frac{\sum_{i,j\in C}\|S_{i}-T_{j}{\|}_{2}}{\sqrt{\left|C\right|}}$$

This score is calculated between each pair of point clouds, resulting in a 2D matrix where is element s,t is the score between point clouds s and t .

### Combining similarity scores

Each similarity calculation method described above (i.e., BLOSUM62, HLAthena, Hamming distance, structural and biochemical) produces a 2D matrix, where element i,j is the similarity between peptides/pHLAs i and j . Each matrix is normalized by subtracting the mean of the matrix and dividing it by the standard deviation. Then, all matrices are summed element-wise, producing a 2D matrix. This matrix provides the similarity score between each pair of pHLAs.

### Clustering

The pairwise similarity scores produced by our method can be used to cluster the peptides. To validate the method, we performed agglomerative clustering using scikit-learn (28). We performed clustering with ward linkage or average linkage. We used agglomerative clustering to create 2 clusters (with parameters n_clusters=2 and distance_threshold=None) to represent the two possible clusters “cross-reactive peptides” and “non-cross-reactive peptides”. We also ran agglomerative clustering to create any number of clusters (with n_clusters=None and distance_threshold=0) and used the result to build a dendrogram to visualize the distance between the different peptides.

We also validated the method using K-nearest-neighbors (KNN) clustering (29). KNN is supervised clustering, meaning the true label of each peptide is known, except the peptide we are attempting to label based on its nearest neighbors. We used k values between 1 and 8 and used KNN to label each peptide based on all the other peptides in the dataset.

### Datasets

We tested our method on five datasets, as explained below. The full list of peptides in each dataset is provided in the Supplementary Data.

#### Dataset 1

Melanoma-associated antigen 3 (MAGE-A3) is an antigen expressed in multiple tumor types, and the MAGE-A3_168–176_ peptide is recognized by a specific T-cell clone. Gee et al. discovered 60 additional peptides that are recognized by the same T-cell clone (30). We use these 60 peptides in addition to MAGEA3 as a positive control for cross-reactivity, for a total of 61 peptides. Negative controls were obtained by searching IEDB for peptides that bind to the same HLA allele (HLA-A*01) (31). 60 peptides were chosen at random, and 59 were chosen for being similar in sequence to the 61 positive controls (i.e., fitting the pattern (EDK) (AGVLIMPFYW) (ED) (PWHST) (MYLK) (DEGN) (AGPVLIMF) (MYFL) (FYL)). The peptide-HLA structures for all 180 peptides were modeled in their docked position to HLA-A*01 using the peptide-HLA modeling tool APE-Gen (16).

#### Dataset 2

The second dataset was obtained from a previous study on T-cell cross-reactivity in HCV peptides (32). This dataset contains 28 peptides, each labeled with a T-cell response level (11 high response, 3 intermediate response, 13 low response, and 1 no response). The pHLA structures were obtained from CrossTope, a curated database of pHLA structures modeled using DockTope (33).

#### Dataset 3

The third dataset is an expansion of the second dataset, containing the 28 HCV peptides and 45 additional peptides (7). The pHLA structures were obtained from CrossTope (33).

#### Dataset 4

The fourth dataset contains 8 Dengue viral peptides, four of which are recognized by the same T-cell, and 4 of which are not (34). The pHLA structures were obtained from CrossTop (33).

#### Dataset 5

The fifth dataset contains 11 peptides, including the cross-reactive pair of peptides HEV-1527 and MYH9-478 and 9 negative controls (35). The pHLA structures were obtained from CrossTope (33).

### PepSim web server

**Figure 1:**
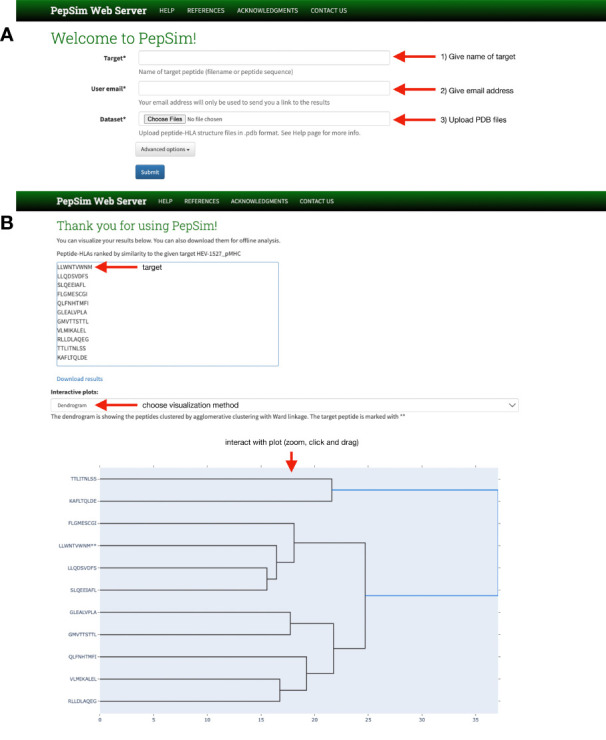
PepSim web server interface. **(A)** The home page allows users to (1) name the peptide they wish to target, either using the amino acid sequence or the file name, (2) give their email address, and (3) upload the PDB files of the peptide-HLAs they with to compare, including the target. A link is sent to the given email address for users to review the results. **(B)** On the results page, users can download the results, view the input peptides in ranked order of similarity to the target, and view interactive plots of either a dendrogram or scatter plot.

The PepSim scoring method is available through a web server interface (see **Figure 1**). Users may upload a dataset in PDB format, including the peptide that they wish to target. The target peptide will be used as the reference for the final ranking of peptides based on similarity. After submission and execution, users can visualize the peptides in a dendrogram based on agglomerative clustering of the peptides based on the similarity scores. The peptides are also visualized in a 2D scatter plot created by using non-metric multidimensional scaling (NMDS) (36). The users also receive a ranked list of the peptides based on their similarity to the given target. If they want to perform offline analysis, including clustering, users can download all the results, which include the 2D array of pairwise similarity scores.

The PepSim web server is implemented using Docker (37), with the backend implemented in Django (38). The submitted jobs are managed by a distributed task queue by Celery (39). The webserver is currently hosted on a virtual machine in the Owl Research Infrastructure Open Nebula (ORION) VM Pool on Rice University Campus.

## Results

### Accurate separation of cross-reactive from non-cross-reactive peptides

**Figure 2:**
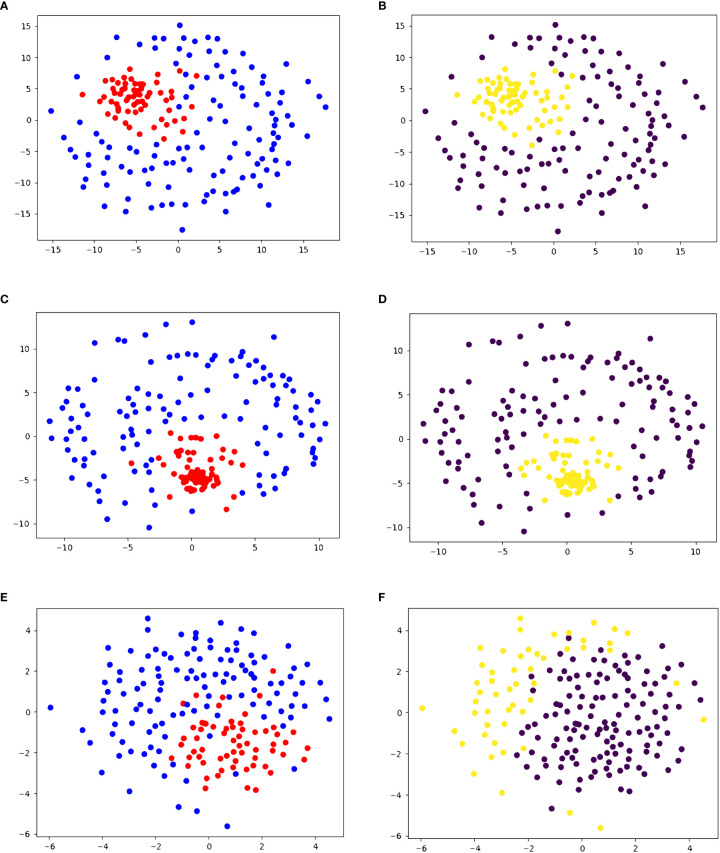
2D embedding of peptides in dataset 1 based on pairwise similarity scores, colored based on **(A, C, E)** true cluster labels and **(B, D, F)** agglomerative clustering results shows the accuracy of PepSim similarity scores. **(A, B)** use the full PepSim similarity score. In **(A)** peptides that are recognized by the same T-cell are red, and the negative decoys are blue. In **(B)**, the yellow cluster corresponds to the cross-reactive peptides. These peptides are clustered using Ward linkage, and the clustering has a sensitivity of 98.36% (1 false negative) and a specificity of 96.64%. **(C, D)** use the sequence analysis similarity score. In **(D)** the peptides are clustered using Ward linkage, and the clustering has a sensitivity of 96.72% (2 false negatives) and a specificity of 97.48% (3 false positives). **(E, F)** use the structural and biochemical similarity scores. In **(F)** the peptides are clustered using Ward linkage and the clustering has a sensitivity of 100% (0 false negatives) and a specificity of 44% (67 false positives). The 2D embedding is created using NMDS (36).

To test the accuracy of our similarity score, we performed the same pipeline on five datasets. On dataset 1, containing 61 peptides that are recognized by the same T-cell and 119 negative decoys, we performed agglomerative clustering with the similarity scores to create two clusters. The resulting clustering had a sensitivity of 98.36% and a specificity of 96.64%. A visualization of the clustering results, as well as the true clustering, can be seen in **Figures 2A, B**. This clustering produced 1 false negative (ASDPMNHYY), and 4 peptides that have not been experimentally determined to produce a T-cell response (ELDPTNMTY, DSDPTGTAY, ELDPDNETY, ELDPNNAVY).

**Figure 3:**
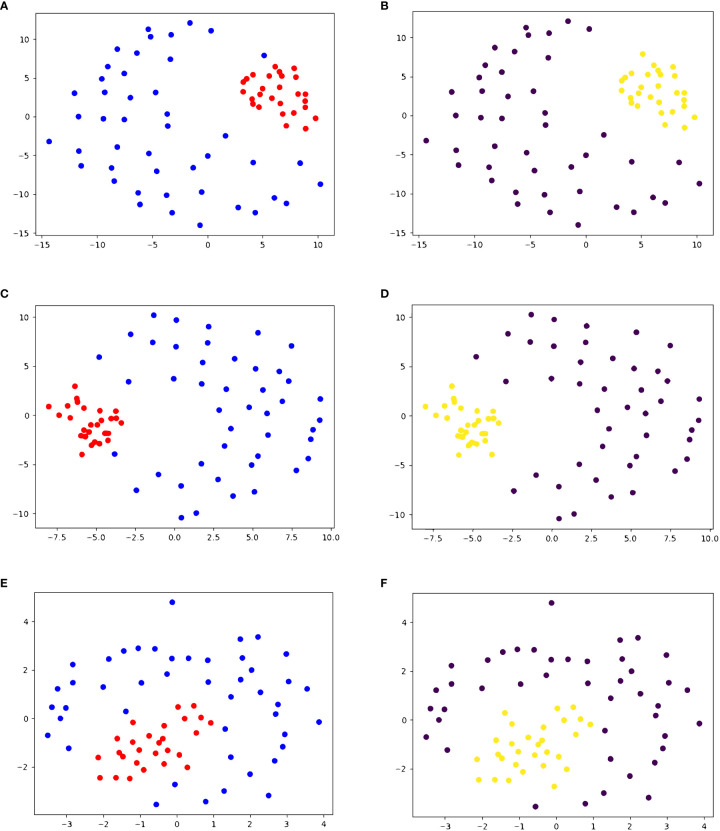
2D embedding of peptides in dataset 3 based on pairwise similarity scores, colored based on **(A, C, E)** true cluster labels and **(B, D, F)** agglomerative clustering result shows the accuracy of PepSim similarity scores. **(A, B)** use the full PepSim similarity score. In **(A)** peptides that are recognized by the same T-cell are red, and the negative decoys are blue. In **(B)**, the yellow cluster corresponds to the cross-reactive peptides. These peptides are clustered using Average linkage, and the clustering has a sensitivity of 100% (0 false negatives) and a specificity of 97.78% (1 false positive). The false positive is a pHLA that was experimentally determined to have no T cell response. **(C, D)** use the sequence analysis similarity score. In **(D)** the clustering also has a sensitivity of 100% (0 false negatives) and a specificity of 97.78% (1 false positive). **(E, F)** use the structural and biochemical similarity scores. In **(F)** the peptides are clustered using Ward linkage, and the clustering has a sensitivity of 100% (0 false negatives) and a specificity of 95.56% (2 false positives). The 2D embedding is created using NMDS (36).

We also performed agglomerative clustering on dataset 3, containing 28 HCV peptides and 45 other viral peptides. One of the HCV peptides causes no T-cell response and the other 27 cause some level of T-cell response. As seen in **Figures 3A, B**, all 28 HCV peptides are clustered in the same cluster, and the decoys are in the other cluster. Given that all but one HCV peptide trigger a T-cell response, this clustering produces only one false positive (G3-18). This exemplifies that our similarity score can differentiate between peptides from different origins, as all the decoys are different viral peptides.

**Figure 4:**
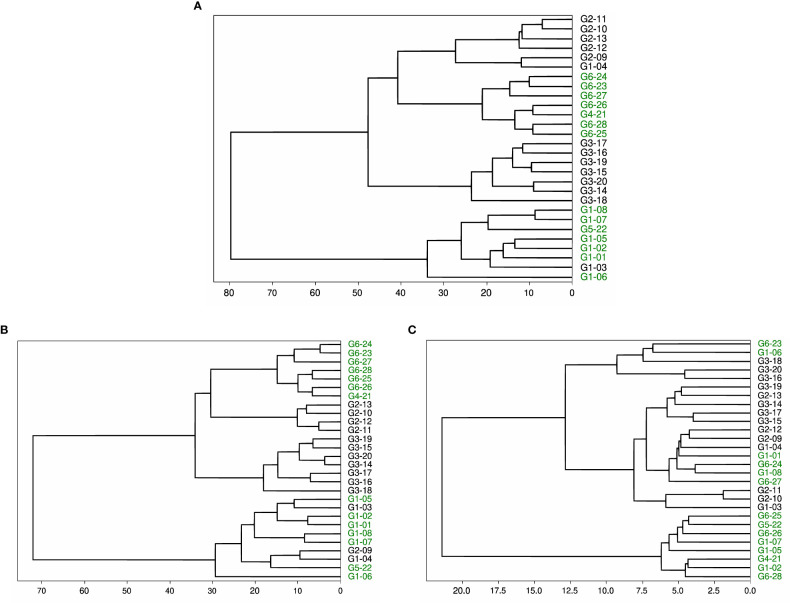
Dendrogram of peptides in dataset 2 based on agglomerative clustering of pairwise similarity scores shows the moderate success of PepSim to differentiate between cross-reactive and non-cross-reactive pHLAs. The peptides that cause a high or intermediate response from the T-cell are labeled in green and clustered into two separate clusters. The peptides that cause a low or no response from the T-cell are in black. In **(A)**, the clustering is based on the entire PepSim similarity score. In **(B)** the clustering is using the sequence similarity scores. In **(C)** the clustering is using the structural and biochemical similarity scores. The best clustering is achieved with the entire PepSim similarity score.

We performed a similar experiment on datasets 2, 4, and 5. In these cases, we did not specify the number of clusters and produced dendrograms for a full visual of the clusters. Dataset 2 contains the same 28 HCV-derived peptides as dataset 3. We see in **Figure 4A** that the final clustering separates the peptides with a high or intermediate T-cell response from most of the peptides with low or no response. The peptides with high or intermediate responses are split into different clusters, but within their separate clusters, they are clustered together. The dendrogram defines two clusters, but if we split the larger cluster based on the dendrogram branches within it, we completely separate the set of high-response peptides from the low and no response groups. The other set of high and intermediate response peptides are in the other cluster in the dendrogram, with one pHLA that produces a low T-cell response (G1-03).

**Figure 5:**
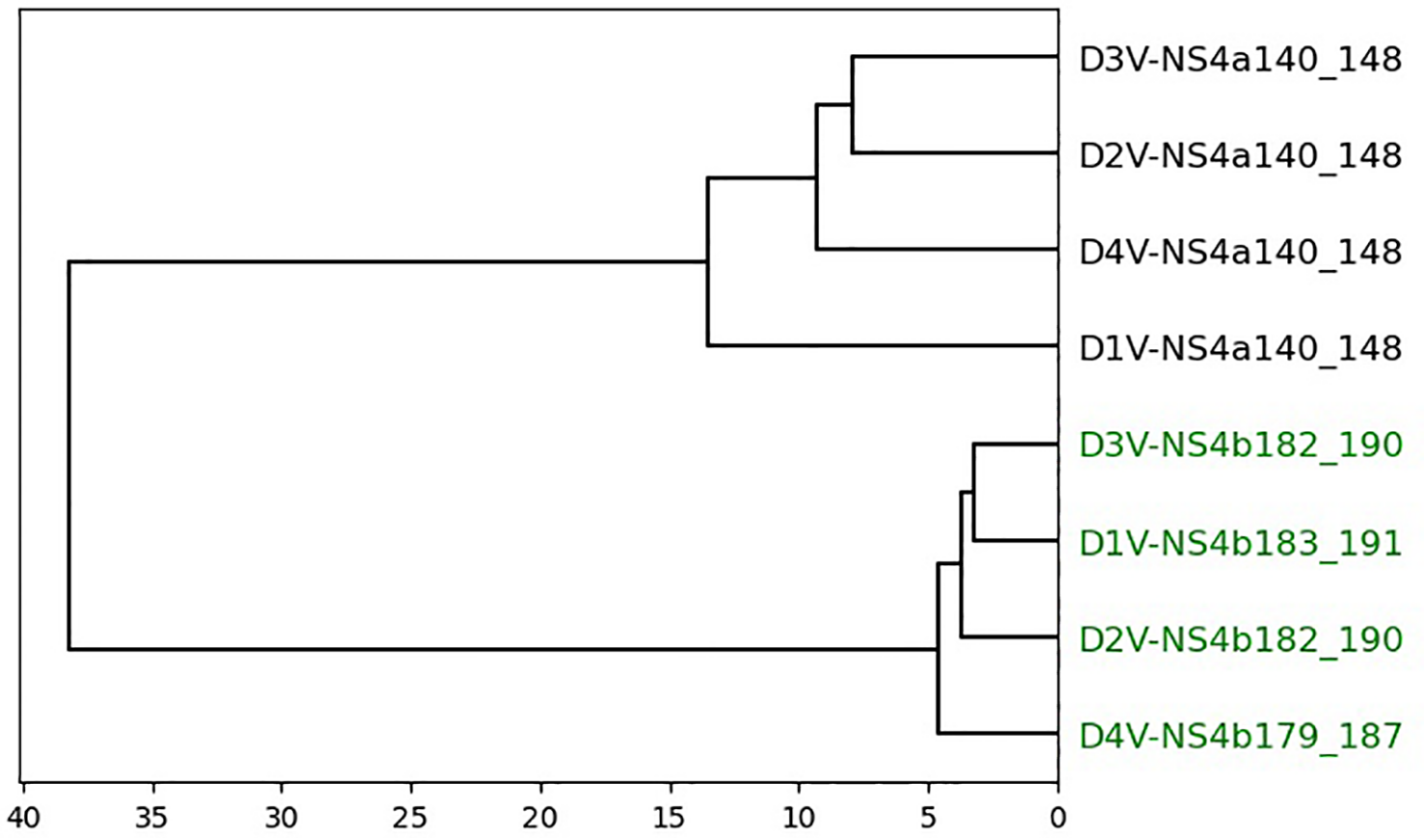
Dendrogram of peptides in dataset 4 based on agglomerative clustering of pairwise similarity scores shows PepSim’s ability to separate cross-reactive from non-cross-reactive pHLAs. The cross-reactive peptides are colored green. Peptides are evenly split into two clusters with all cross-reactive peptides in one cluster and all non-cross-reactive peptides in the other. These peptides are clustered using Ward linkage, and Average linkage produces the same clusters.

**Figure 6:**
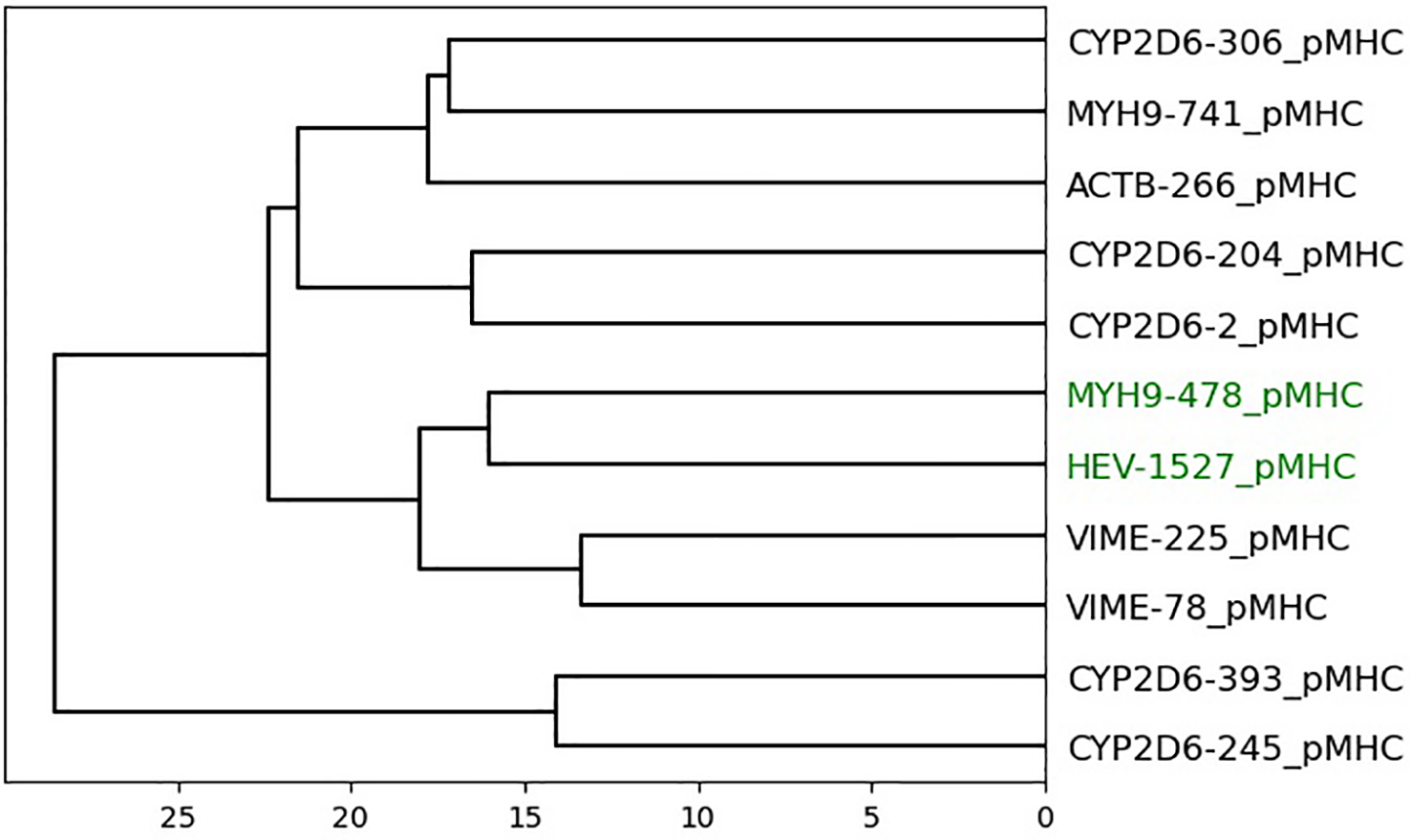
Dendrogram of peptides in dataset 5 based on agglomerative clustering of pairwise similarity scores shows PepSim’s ability to separate cross-reactive from non-cross-reactive pHLAs. The peptides that are recognized by the same T-cell (HEV-1257 and MYH9-478) are labeled in green and are in the same branch of the dendrogram. If you select the target peptide HEV-1527, then the closest peptide is MYH9-478, and vice versa.

Datasets 4 and 5 are smaller datasets with a low number of cross-reactive peptides. Dataset 4 shows a complete separation of cross-reactive and non-cross-reactive peptides, as seen in **Figure 5**. The cross-reactive peptides are very similar in sequence. Dataset 5 contains two cross-reactive peptides: HEV-1527 and MYH9-478. As seen in **Figure 6**, HEV-1527 and MYH9-478 are not separated into a different cluster from the negative decoys, but they are on the same branch of the dendrogram. If you select the target peptide HEV-1527, then the closest peptide is MYH9-478, and vice versa. Given that this method would start with a target peptide, then we can accurately identify the cross-reactive peptide from this dataset.

### Comparison with predictions in the literature

In 2015, Mendes used multivariate statistical methods to perform structure-based prediction of T-cell cross-reactivity among the 28 viral peptides in dataset 2 (8). Using their method, they clustered the peptides into two distinct clusters. The peptides that trigger a high T-cell response are placed in one cluster and the peptides with a low response are in the other cluster. One of the peptides with an intermediate response in the cluster with the high response peptides, and the other two are with the low response peptides. In contrast, with our method, the peptides with a high response are split into two different clusters, and the peptides with an intermediate response are clustered with the peptides with a high response.

Dataset 3, being an expansion of dataset 2, was previously studied in 2011 by Antunes et al. (7) Only 10 of the HCV peptides were included in the dataset (A0201_0031, A0201_0051, A0201_0052, A0201_0053, A0201_0054, A0201_0055, A0201_0056, A0201_0057, and A0201_0058), along with 45 other peptides. All 10 of the HCV peptides were clustered together, along with five other peptides not derived from HCV (A0201_0014, A0201_0083, A0201_0076, A0201_0095, and A0201_0073). In contrast, our method results in all the HCV peptides clustered together with only one false positive, A0201_0033, which is an HCV peptide that causes no T-cell response. We can also look at our results on dataset 2, where peptides A0201_0051-58 are clustered together with no other clusters, and A0201_0031 is in the other cluster.

### The effects of structural analysis

To examine the effectiveness of the structural and biochemical analysis, we repeated the experiments with two variations of the similarity score. We defined one variation of the score to use only sequence analysis, and the other variation to use only the structural and biochemical analysis.

In dataset 1, when we use only sequence analysis, the final agglomerative clustering (using Ward linkage) has a sensitivity of 96.72% (2 false negatives) and specificity of 97.48% (3 false positives), as seen in **Figures 2C, D**. Compared to the similarity with the structural and biochemical analysis included, there is one more false negative and one less false positive when we remove the structural and biochemical analysis.

When we use the similarity score calculated from only the structural and biochemical analysis, the sensitivity increases to 100%, but the specificity decreases to 44%, as seen in **Figures 2E, F**.

We also used K-nearest-neighbors to determine the effectiveness of the similarity score. As seen in **Table 1**, we achieved the highest sensitivity and specificity when we use all the components of the similarity score (sensitivity of 100% and specificity of 98.3%). We also achieve high accuracy with the variations of the similarity score, including using only the structure and biochemical analysis

**Table 1:** Accuracy of leave-one-out KNN cross-validation on Dataset 1.

k	Complete score		Only sequence		Only structure/biochemical	
	sensitivity	specificity	sensitivity	specificity	sensitivity	specificity
1	1.000	1.000	1.000	1.000	1.000	1.000
2	1.000	1.000	1.000	1.000	0.918	1.000
3	1.000	0.966	1.000	0.958	0.951	0.933
4	1.000	0.983	1.000	0.966	0.885	0.953
5	1.000	0.924	1.000	0.882	0.902	0.875
6	1.000	0.958	0.984	0.899	0.902	0.924
7	1.000	0.832	1.000	0.824	0.902	0.857
8	1.000	0.874	1.000	0.849	0.902	0.916

In dataset 3, removing the structural and biochemical analysis from the similarity score does not change the final clustering when using Ward or Average linkage, as seen in **Figures 3C, D**. Similarly, using only the structural and biochemical analysis results in one additional false positive when using Ward linkage (A0201_0014). **Figures 3E, F** shows the 2D embedding of the peptides based on the structural and biochemical similarity score and compared to **Figures 3A, B** the clusters are not as separated.

We also used K-nearest-neighbors to determine the effectiveness of the similarity score. As seen in **Table 2**, we achieved the highest sensitivity and specificity when we use all the components of the similarity score (sensitivity of 100% and specificity of 95.6%). We also achieve high accuracy with the variations of the similarity score, including using only the structure and biochemical analysis.

**Table 2:** Accuracy of leave-one-out KNN cross-validation on Dataset 3.

k	Complete score		Only sequence		Only structure/biochemical	
	sensitivity	specificity	sensitivity	specificity	sensitivity	specificity
1	1.000	1.000	1.000	1.000	1.000	1.000
2	1.000	1.000	1.000	1.000	1.000	1.000
3	1.000	0.933	1.000	0.911	1.000	0.822
4	1.000	0.956	1.000	0.933	1.000	0.911
5	1.000	0.867	1.000	0.867	1.000	0.778
6	1.000	0.867	1.000	0.889	1.000	0.867
7	1.000	0.844	1.000	0.867	1.000	0.800
8	1.000	0.844	1.000	0.889	1.000	0.867

justification=centering

Dataset 2 provides more interesting results. When the structural and biochemical analysis is removed from the similarity score, the dendrogram clustering shows that the high and intermediate responders are still separated into two different clusters, as seen in **Figure 4B**. One of the clusters has three false positives compared to the one false positive in **Figure 4A**.

When using only structural and biochemical analysis, there is one cluster of all peptides with a high or intermediate T-cell response, and the other peptides with a high response are separated from each other (**Figure 4C**). Therefore, the clusters are less accurate when only using the structural and biochemical analysis when compared to the complete score. However, the peptides derived from different viral genotypes are clustered together.

We also used K-nearest-neighbors to determine the effectiveness of the similarity score. As seen in **Table 3**, we achieved the highest sensitivity and specificity when we use all the components of the similarity score (sensitivity of 100% and specificity of 92.9%). We also achieve high accuracy with the variations of the similarity score, including using only the structure and biochemical analysis.

**Table 3:** Accuracy of leave-one-out KNN cross-validation on Dataset 2.

k	Complete score		Only sequence		Only structure/biochemical	
	sensitivity	specificity	sensitivity	specificity	sensitivity	specificity
1	1.000	1.000	1.000	1.000	1.000	1.000
2	0.929	1.000	1.000	1.000	0.929	1.000
3	1.000	1.000	1.000	1.000	0.929	1.000
4	1.000	1.000	0.929	1.000	0.714	1.000
5	1.000	0.929	1.000	0.929	1.000	0.929
6	1.000	0.929	1.000	0.929	0.929	1.000
7	1.000	0.857	1.000	0.857	1.000	1.000
8	1.000	0.929	1.000	0.929	0.857	1.000

Datasets 4 and 5 show little change when removing the structural and biochemical analysis and when removing the sequence analysis. In dataset 4, the four cross-reactive peptides and four non-cross-reactive peptides are separated into different clusters with 100% accuracy. In dataset 5, again HEV-1527 and MYH9-478 are not separated into a different cluster from the negative decoys, but they are on the same branch of the dendrogram.

## Discussion

T-cell cross-reactivity can cause devastating side effects in T-cell-based cancer immunotherapy, therefore it is of vital importance that we predict cross-reactivity when choosing immunotherapy targets. Here we proposed a scoring method to determine the similarity between peptide-HLA complexes to predict T-cell cross-reactivity. The metric we used here incorporates several methods of comparing peptides and peptide-HLAs including sequence, structure, and biochemical analysis. Peptide-HLAs that are more similar to each other are more likely to trigger an immune response from the same T-cell (7–9), thus our score can be used to predict T-cell cross-reactivity.

When we run our method on dataset 1, the agglomerative clustering accurately separates the cross-reactive peptides from the decoys with 1 false negative and 4 false positives. In this case, the decoys have not been experimentally validated, so what we call a false positive may actually be cross-reactive and is a good candidate for further experimentation. We successfully separate most of the cross-reactive peptides from the negative decoys that are similar in sequence to the cross-reactive peptides. We have partial success when we use dataset 2. We are not able to reproduce the previous results of Mendes and colleagues (8), but their method was specialized for dataset 2. They had previous knowledge of the TCR-interaction contacts, so specific areas of the peptide-HLAs were selected for analysis. Our method is designed to work on any dataset of class I peptide-HLAs. As shown in our results on dataset 2, the peptides from genotype 6 are clustered together, and the peptides from genotype 1that trigger a T-cell response are also clustered together. Interestingly, when we use only the structural and biochemical analysis in the score, we get one cluster of peptides from multiple genotypes that are all cross-reactive and the other cross-reactive peptides are spread out in the other cluster. This clustering of different genotypes does not occur when we use only sequence analysis or when combining the sequence and structure analysis. Therefore, we can assume that the structural and biochemical analysis is recognizing similarities between peptide-HLAs that the sequence analysis is missing. Our KNN analysis has high sensitivity and specificity regardless of the score composition. In dataset 3, an expansion of dataset 2, we successfully separate the HCV-derived peptides from the negative decoys. Lastly, in the small datasets 4 and 5, the cross-reactive peptides are clustered together, showcasing how our method works on smaller datasets.

In this paper, we have presented a comparison to a previous method of predicting T-cell cross-reactivity *via* statistical analysis of the peptide-HLA structural features (8). This previous method has better results compared to our score, but each is only presented on a single dataset and specialized to that dataset. It is also based on 2D image analysis, and therefore only partially accounts for the structural and biochemical features of the pHLA complexes. The structure is simplified from 3Dto a 2D image, whereas in our method we use the 3D structure. We have also analyzed the effects of structural and biochemical information on the accuracy of our tool. We find that structural and biochemical analysis is useful in determining the similarity between peptide-HLA complexes, but peptide sequence analysis is also vital to accurately determining peptide-HLA similarity.

There are other methods of cross-reactivity prediction that can be potentially compared to PepSim. JanusMatrix is part of EpiVax’s proprietary immunogenicity screening kit and thus cannot be freely compared to PepSim. In the original study, JanusMatrix is used to find potential cross-reactivity to defined T-cell epitopes (one from HCV and one from influenza), but the authors only provide the number of cross-reactive hits and not the peptides that are potentially cross-reactive (12). Therefore, a comparison is difficult. Expitope 2.0 is available as a web server where the user inputs a single peptide and receives a calculated cross-reactivity index chart. In the original study, the authors show that Expitope successfully predicts the cross-reactivity between MAGEA3 and the Titin-derived peptide (15). In this paper, we have shown that PepSim successfully predicts cross-reactivity to MAGEA3. RACER is an energy model for predicting TCR-pMHC binding affinity and can be used to predict T-cell cross-reactivity. RACER is shown to accurately predict TCR recognition rates when tested on datasets of class II MHCs, and thus we cannot compare PepSim, which was designed for class I MHCs.

As we have shown, our method applies to datasets of different sizes and content. Dataset 1 includes cancer peptides and self-peptides, and the other datasets consist of different viral peptides. Each dataset is also of a different size, but each experiment produces an accurate clustering of the peptides. Also, our method is T-cell independent, meaning no information on the TCR including sequence or structure is necessary to compute the similarity score. Our score provides a likelihood of triggering a cross-reactive response, based on the driving impact of the pMHC similarity. However, experimental measurements of cross-reactivity between these pHLAs might provide different results depending on the specific T-cell clone that is used in the experiments. Our ability to perfect the methods presented in this paper is limited by data availability, as we are achieving high classification performance on the presented datasets. Thus, it is likely that similar methods will be able to build on this work to achieve greater performance as more T-cell cross-reactivity data becomes available.

In terms of usability, Pepsim is available as a web server. PepSim takes the PDB files of each pHLA structure. The input files can be from multiple different sources, such as experimentally determined structures from the Protein Data Bank (11) or computational modeling software such as APE-Gen (16) or DockTope (17). With modeling software, researchers can generate structures for any number of peptide-HLA pairs. Given computational cost, we recommend using a dataset of at most 500 peptide-HLAs. PepSim is designed to be TCR independent, so it can be used when the peptide residues that are important to TCR recognition are unknown. However, we recognize that knowing the important residue positions would potentially improve PepSim’s predictions, so a user of the web server has the option of specifying weights for the different peptide residue positions. In addition, although PepSim performs well in this study without needing to change the weights of the different sub-scores, the web server user can input their own sub-score weights. Additionally, the user defines a “target” pHLA, and PepSim outputs a ranked list of the input pHLAs in order of likelihood of cross-reactivity with the target pHLA. PepSim also generates the 2D score matrix that the user can use for further analysis, including clustering, and PepSim generates a dendrogram with the results of hierarchical clustering.

## Conclusion

PepSim helps to fill a gap in the existing methods for predicting T-cell cross-reactivity. Previous attempts to incorporate structural features into cross-reactivity analysis were hindered by the lack of structures and the high computational demand of sampling methods, but we can overcome these limitations by relying on fast modeling through APE-Gen, and efficient algorithms for geometrical comparisons. In a large dataset (Dataset 1), we were able to accurately separate cross-reactive from non-cross-reactive peptides. Our method can also be generalized, as demonstrated in other smaller datasets (Datasets 2-5). Additionally, our method does not depend on the size and content of datasets and can be used in a T-cell-independent manner. PepSim is available as a webserver at pepsim.kavrakilab.org.

## Data availability statement

The original contributions presented in the study are included in the article/**Supplementary Material**. Further inquiries can be directed to the corresponding author.

## Author contributions

SH-S, MR, LK, and GL contributed to the conception and design of the study. SH-S and JS developed the methodology and software. SH-S and MR curated data. SH-S wrote the manuscript, performed the experiments, and created the web server. LK, DA, and GL were responsible for the supervision and project administration. LK was responsible for funding acquisition. All authors contributed to the article and approved the submitted version.
